# Lack of association between apolipoprotein C3 gene polymorphisms and risk of coronary heart disease in a Han population in East China

**DOI:** 10.1186/1476-511X-10-200

**Published:** 2011-11-04

**Authors:** Juan Yu, Jingjing Huang, Yan Liang, Baodong Qin, Su He, Jing Xiao, Huimin Wang, Renqian Zhong

**Affiliations:** 1 Department of Laboratory Medicine, Changzheng Hospital, Second Military Medical University and Clinical Immunology Center of PLA, 415 Feng Yang Road, Shanghai 200003, People's Republic of China; 2 Center of Laboratory Medicine, Affiliated Hospital, Nantong University, 20 Xi Si Road, Nantong 226001, People's Republic of China; 3 Institute of Public Health, Nantong University, 9 Se Yuan Road, Nantong 226001, People's Republic of China

**Keywords:** Apolipoprotein C3, Coronary heart disease, Polymorphism, Hypertriglyceridemia

## Abstract

**Background:**

Several polymorphisms in the apolipoprotein C3 (APOC3) gene have been found association with hypertriglyceridemia(HTG), but the link with coronary heart disease(CHD) risk between ethnicities was still controversial. Among them, reseachers paid more attentions to the promoter polymorphisms T-455C and C-482T because both of them located in insulin-responsive element (IRE) and insulin was thought to exert its action by down-regulating APOC3 gene expression. The aim of this study was to investigate the association of the two polymorphisms of APOC3 with CHD in a Han population in East China.

**Methods:**

TaqMan SNP Genotyping Assays were carried out to detect the genotypes of APOC3 gene, including the T-455C and C-482T, in 286 subjects with CHD and 325 controls without CHD. The levels of serum lipid profiles were also detected by biochemical methods.

**Results:**

There was no difference of genotype frequencies and allele frequencies between the CHD population and the controls(P > 0.05). Compared with the most common genotype -455TT or -482CC, the variants had neither significantly increased CHD risk, nor the lipid variables showed any statistically relevant differences in the research population. The adjusted OR of CHD were 5.67 [0.27-18.74] and 0.75 [0.20-2.73] in carriers of the APOC3 -455C and -482T variants, respectively(P > 0.05). There was also no significant difference in APOC3 haplotype distribution in CHD and controls, but there was a strong linkage disequilibrium between T-455C and C-482T with D' = 0.9293, 0.8881, respectively(P < 0.0001).

**Conclusions:**

Our data did not support a relationship between the two polymorphisms of APOC3 gene and risk of CHD in the Han population in East China.

## Background

Both the genetic and environmental factors contribute to the development of coronary heart disease (CHD) which is becoming the leading cause of death in many countries of the world including China. The metabolism of circulating particles such as triglyceride-rich lipoprotein (TRL) was strongly influenced by their content in apolipoprotein C3 (APOC3), a component that inhibits the hydrolysis of these paticles by lipoprotein lipase, and APOE-mediated hepatic uptake of them. By far, data from both clinical evidence [1,2] and mechanism research results [3,4] supported the role of APOC3 in CHD and artherosclerosis(AS), suggesting that the APOC3 was a strong independent risk factor for CHD.

Different genetic and acquired factors impact serum APOC3 concentrations. Five polymorphisms have been found in the APOC3 gene promoter. Among them, two promoter polymorphisms T-455C and C-482T have been studied more extensively because they just located on the insulin-responsive element (IRE) in APOC3 gene. Insulin exerted its action by down-regulating APOC3 gene expression transcriptionally, however, the presence of mutant sequences seemed to reduce the inhibitory effect of the hormone [5].

Patients with type 2 diabetes mellitus(T2DM) increased levels of circulating APOC3, and it has been linked to enhanced β-cell apoptosis [6]. Recently we reported that the T-455C or C-482T variant on the APOC3 gene promoter contributed to an increased risk of HTG in Chinese Han health population, however, no relationship between the two polymorphisms and HTG in T2DM patients was found [7]. Insulin resistance state in T2DM may mask the effect of a common APOC3 haplotype on the risk of HTG. A large body of clinical studies have demonstrated that increased serum triglyceride concentrations were positively associated with risk for CHD [2,8,9], and low APOC3 levels were associated with reduced CHD risk [10,11]. Although several studies have supported an association between these two polymorphisms and CH#1 [12], others have failed to confirm the relationship [13,14]. Considering the complex of CHD and race difference, what condition is in china? In present study, we investigated the distribution of the two polymorphisms and the relationship to the susceptibility of CHD in the Han Population in East China.

## Methods

### Study population

we selected a total of 611 unrelated adult subjects of both sexes, 286 unrelated Chinese patients (214 males, 72 females, age 56.30 ± 11.57 years) with significant coronary stenosis by cardiovascular angiography(according to stenoss ≥ 50% at least 1 coronary artery)were recruited from Department of cardiolog of Changzheng Hospital, Second Military Medical University of China. 325 controls were selected via health-screening at the Physical Examination Centre or CHD-free of heart disease patients at the same hospital (172 males, 153 females, age 55.79 ± 12.40 years). All subjects were of Han ethnicity in East China.

The informed consent was obtained from subjects who agreed to participate in the study, and the study had the approval of the hospital ethics committee.

### Biochemical analysis

The blood sample after 12 hours fasting was collected in tube for measuring. TG, total cholesterol (TC), HDLC and LDLC were measured using the enzymatic method, and APOA1 and APOB were determined by immunonephelometry, and they all measured by automatic biochemistry analyzer (Abbott Aeroset 2000, USA). APOC3 was also measured by automatic biochemistry analyzer (Hitachi 7020, Japan). Samples for APOC3 were measured by a turbidimetric immunoassay, and the reagents were obtained from Sekisui medical co.(Japan) with a precipitant reagent [phosphotungstic acid 0.55 mmol/l and magnesium chloride (MgCl_2_) 25 mmol]. HDL-APOC3 was assayed similarly in the supernatant. TRL-APOC3 was calculated by substracting HDL-APOC3 from total APOC3.

### Mutation analysis

**Figure 1:**
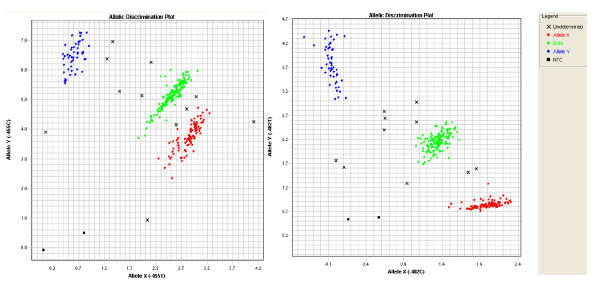
**TaqMan SNP Genotyping figure of indicated promoter polymorphisms in APOC3 gene**. Left: T-455C, right: C-482T.

TaqMan SNP Genotyping Assays were carried out to detect the genotypes of APOC3 gene, including the T-455C and C-482T, in 286 subjects with CHD and 325 controls. There was 20 missing data of genotype due to the template reason(17 in CHD, and 3 in controls). A 2 ml whole-blood sample containing EDTA was collected for each subject. Genomic DNA was extracted using a Genomic DNA Kit(Axygen Biotechnology, shanghai, China) according to the manufacturer's protocol and store the DNA samples in a -20°C freezer until use. Two valid SNPs rs2854116(T-455C) and rs2854117(C-482T) in the APOC3 gene that are found to be associated with HTG were genotyped. Genotyping was performed applying a TaqMan assay that incorporates minor groove-binding probe technology. A fluorogenic probe consisting of an oligonucleotide labeled with both fluorescent reporter and quencher dyes was included. The primers of rs2854116 are AGAGCTCAGCCCTGTAACCA(forward) and GGGCTTCTTCAGACTTGAGAACAA(reverse), and the probes were VIC-ACTCCAAACATCCCCC-MGB and FAM-CTCCAAACACCCCCC-MGB. The primers of rs2854117 were same as rs2854116, but the probes were VIC-CAGAAGACCGGGCATC-MGB and FAM-CAGAAGACCAGGCATC-MGB. All PCR reactions were initially run in a final volume of 5 μl, comprising of 2.5 μl TaqMan genotyping master mix, 0.25 μl TaqMan SNP genotyping assay mix (40×), 1 μl DNA samples, 1.25 μl water. All PCR reactions was carried out in the ABI 7900 with 384 wells, consisting of an initial step of 95°C for 5 min, followed by 40 cycles: denaturation at 95°C for 15 sec, annealing at 60°C for 60 sec. Amplicon size was 115 bp. Genotyping or allele calling was carried out with the ABI Prism 7900HT genetic detection system: SDS 2.3 software. A graph on the computer would show the results of the allelic discrimination plot by automatic allele calling. The horizontal axis indicated an wild allele XX homozygote, the vertical axis indicated mutant allele YY homozygote, and the diagonal was allele X/Y heterozygote(Figure 1). When automatic genotyping was not well done, re-testing, or direct sequencing, until got the results. To validate the SNP genotyping assays, 5% of the genotyped samples were randomly selected for duplication accuracy with a direct sequencing protocol(Sangon Biotech, Shanghai, China) and all of the data for the TaqMan SNP Genotyping Assays and direct sequencing analyses showed perfect concordance.

### Statistical analysis

Baseline characteristics were shown as mean ± SD. Due to plasma Lipid concentrations had a skewed distribution, the median and interquartile ranges (Q:difference of Q25 and Q75) were presented: M ± Q. The association between genetic polymorphisms and the prevalence of CHD was assessed using chi-square tests. Odds ratio was determined by comparing each polymorphism (heterozygous, homozygous) to wild type and the 95% confidence intervals were calculated. Observed frequencies in the CHD and controls were compared to the expected frequencies (Hardy-Weinberg equilibrium) by chi-square test. The distribution of genotype and allele frequencies in CHD and controls were analyzed by chi-square test too. The difference of lipid levels in different genotypes in CHD and controls was evaluated by one-way ANOVA after rank transformation when necessary. Linkage disequilibrium was calculated by Arlequin population genetics analysis software showed in value of D'. All computations were performed by using the stata 8.0 or SAS 9.13 statistical software. Levels of significance were defined as *P *< 0.05.

## Results

Genotype and allele frequencies of the APOC3 polymorphic variants for CHD and controls were described in Table 1. The allele frequencies of the rs2854117 (-482T) and rs2854116 (-455C) APOC3 alleles were 0.424 and 0.420 in CHD, 0.407 and 0.410 in controls, respectively. Three polymorphisms were in the Hardy-Weinberg equilibrium in relevant groups (data not shown). There was no significant difference of the genotype and allele distribution between CHD and controls(*P *> 0.05).

**Table 1:** Distribution of genotype and allele frequencies of APOC3 in CHD and controls

	**CHD**(**%)**	control(%)	**χ**^**2**^	P
-455 Genotype frequency				
-455TT	90(33.58)	112(34.67)		
-455TC	131(48.88)	157(48.61)		
-455CC	47(17.54)	54(16.72)	0.11	0.95
-455 Allele frequency				
-455T	311(58.00)	381(59.00)		
-455C	225(42.00)	265(41.00)	0.11	0.74
-482 Genotype frequency				
-482CC	89(33.21)	11(34.67)		
-482CT	131(48.88)	159(49.23)		
-482TT	48(17.91)	52(16.10)	0.38	0.83
-482 Allele frequency				
-482C	309(57.60)	383(59.30)		
-482T	227(42.40)	263(40.70)	0.32	0.57

Numbers in parentheses indicate each frequency of genotype or allele.

For the purpose of estimating the impact of the polymorphisms on APOC3 and lipid levels and other accompany complications, genotype-phenotype analysis was performed with data from the CHD and controls. Compared to the wide type -455TT or -482CC, none of the lipid variables and incidence of other accompany complications analyzed showed any statistically relevant differences according to the variant APOC3 promoters in the study population(Table 2).

**Table 2:** Biochemical parameter levels in study population, according to the APOC3 T-455C and C-482T genotypes

**Parameters**	**-455 Genotypes**			**-482 Genotypes**		
	**-455TT**	**-455TC**	**-455CC**	**-482 CC**	**-482CT**	**-482TT**
	
Age (years)	55.64 ± 16.76	55.54 ± 16.21	55.48 ± 14.84	55.22 ± 16.60	55.56 ± 16.03	55.39 ± 15.11
Male sex (%)	65.7	62.5	60.4	66.5	61.0	63.0
TC (mmol/L)	3.92 ± 1.16	4.00 ± 1.18	3.84 ± 1.28	3.91 ± 1.18	3.99 ± 1.15	3.87 ± 1.33
TG (mmol/L)	1.29 ± 1.05	1.35 ± 1.17	1.50 ± 1.20	1.25 ± 0.98	1.38 ± 1.25	1.47 ± 1.12
HDLC (mmol/L)	1.12 ± 0.35	1.14 ± 0.38	1.10 ± 0.28	1.13 ± 037	1.13 ± 0.39	1.09 ± 0.27
LDLC (mmol/L)	2.34 ± 0.94	2.47 ± 0.80	2.40 ± 0.83	2.34 ± 0.90	2.44 ± 0.84	2.48 ± 0.85
APOAl (g/l)	1.3 ± 0.57	1.36 ± 0.58	1.29 ± 0.54	1.35 ± 0.57	1.33 ± 0.57	1.28 ± 0.56
APOB (g/l)	0.84 ± 0.29	0.85 ± 0.31	0.86 ± 0.31	0.84 ± 0.27	0.85 ± 0.31	0.88 ± 0.35
APOAl/APOB	1.69 ± 0.43	1.83 ± 0.60	1.68 ± 0.44	1.69 ± 0.40	1.84 ± 0.63	1.68 ± 0.42
APOC3(mg/dl)	8.10 ± 4.1	8.4 ± 4.5	7.95 ± 3.3	8.4 ± 4.3	8.1 ± 4.5	8.3 ± 3.1
HDL-APOC3 (mg/dl)	5.2 ± 2.4	5.4 ± 2.2	5.4 ± 1.8	5.2 ± 2.2	5.4 ± 2.2	5.4 ± 2.0
TRL-APOC3 (mg/dl)	3.35 ± 3.25	2.8 ± 2.6	3.5 ± 3.4	3.2 ± 3.1	3.0 ± 2.8	3.35 ± 3.15
T2DM (%)	20.1	20.56	26.73	18.2	21.1	28.0
Hypertension (%)	44.6	44.9	51.5	41.9	45.7	54.0
smoker(%)	44.9	45.5	38.4	44.7	43.5	43.2
drinker(%)	28.3	21.2	26.0	28.0	20.7	28.4

Data were shown as mean ± SD or %; skewed variables were M ± Q. M: median, Q: inter-quartile range, difference of Q25 and Q75.

To estimate the CHD risk associated with APOC3 genotypes, logistic regression analysis was performed. Crude and adjusted odds ratios for CHD related to APOC3 -455 and 482 genotypes are shown in Table 3. The polymorphisms of APOC3 gene promoter were not associated with a significantly increased risk of CHD in the study population.

**Table 3:** Odds Ratios for CHD according to APOC3 genotypes in the study population

	Unadjusted Model		adjusted Model	
	
APOC3 genotypes	Odds Ratio	*P *value	Odds Ratio	*P *value
	(95%CI)		(95%CI)	
-455TT	1		1	
-455TC (-455TT as reference)	1.00 (0.66-1.51)	0.98	1.6 (0.20-6.81)	0.16
-455CC (-455TT as reference)	1.06(0.61-1.88)	0.82	5.67 (0.27-18.74)	0.26
-482CC	1		1	
-482CT (-482CC as reference)	1.00 (0.66-1.51)	1.00	0.87 (0.12-6.25)	0.89
-482TT (-482CC as reference)	1.06 (0.60-1.87)	0.85	0.75 (0.20-2.73)	0.55

By multiple logistic regression analysis, The multiple logistic-regression model was adjusted for age, gender, presence of hypertension, T2DM, drinking, smoking and all listed lipid leveles. CI, confidence interval. P values were for the overall comparison among subjects with a given polymorphism and were calculated by chi-square analysis.

To estimate the CHD risk associated with APOC3 haplotypes, Arlequin population genetics analysis software was applied(Table 4). No relationship was found between haplotypes of APOC3 -455 and -482 and CHD in the study population, but there was a strong linkage disequilibrium between T-455C and C-482T with D' = 0.9293, 0.8881, respectively(Table 5).

**Table 4:** APOC3 haplotype distribution in CHD and controls

-455	-482	CHD n = 269	Controls n = 322	χ2	p
1	1	0.5109	0.5239	2.11	0.55
1	2	0.1842	0.1486		
2	1	0.0821	0.0678		
2	2	0.2228	0.2507		

1 and 2 stand for common and rare alleles, respectively.

**Table 5:** linkage disequilibrium analysis of T-455C/C-482T

Group	n	D'	**r**^**2**^	P
CHD	269	0.9293	0.1723	< 0.0001
Controls	322	0.8881	0.2775	< 0.0001
Total	591	0.9085	0.2251	< 0.0001

## Discussion

APOC3, a 79 amino acid glycoprotein synthesized mainly in the liver and to a lesser degree in the intestine, which acted as a constituent of TRL particles and functioned as a key regulator of serum triglyceride levels, inhibiting the lipoprotein lipase-induced hydrolysis of those particles and interfering with liver receptor-mediated lipoprotein uptake. It played a key role in determining the levels in the circulation of potentially atherogenic VLDL and small dense LDL [15]. The results of large clinical studies have indicated that sera APOC3 concentrations were a better predictor of risk for the development and progression of CHD than the traditionally measured TG levels [1,8]. APOC3 could also irritate several processes involved in atherogenesis and vascular inflammation. APOC3 stimulated blood-born monocytes and endothelial cells to activate protein kinase C (PKC) and nuclear factor κB (NF-κB) and expression of endothelialproduce vascular cell adhesion molecule-1 (VCAM-1) and intracellular adhesion molecule-1(ICAM-1), and recruitment of monocytes to the vascular wall [16-18]. APOC3 could also activate insulin-resistance pathways in endothelial cells causing endothelial dysfunction [19] and stimulate adipocytes to produce cytokines such as monocyte chemoattractant protein (MCP) 1 and interleukin (IL)6 and suppresses their production of adiponectin [20].

APOC3 was the first lipid-associated gene to be linked by a common polymorphism to hypertriglyceridemia [21]. Genetic variation in the APOC3 gene, particularly in the promoter region, was associated with an increased risk of HTG [5], metabolic syndrome(MS)[22-24] and CHD [12] in some race population. Association between promoter SNPs of APOC3 and plasma triglyceride levels have been consistently reported. The relationship with CHD, however, was more controversial, with some studies indicating a possible link with genetic variability at the locus [12] and others not confirming those associations [13]. In the present study, the association between APOC3 SNPs and CHD was assessed by comparing the SNPs distribution in CHD and controls without CHD. We demonstrated the relationship between the two polymorphisms T-455C and C-482T and CHD in a randomly selected population sample of Han Population in East China. However, neither APOC3 T-455C nor C-482T SNPs were clearly associated with CHD. Together with previous reports, these results indicated that, although APOC3 T-455C or C-482T variant has a vital impact on plasma triglyceride concentration, this effect did not increase significantly CHD risk.

Further haplotype analyses also did not found any significant frequency difference between CHD and controls, but found there was a strong linkage disequilibrium between T-455C and C-482T.

Ruiz et al reported that cardioprotective effects of promoter common allele -455T or -482C of APOC3 gene became dysfunctional in abdominal obesity and hyperglycemia in nonfatal MI [25]. Their research suggested that the effect of the APOC3 -455C and -482T haplotype on the risk of nonfatal MI was only evident in lean normoglycemic subjects. In the previous study we also found that the effect of these two variant APOC3 genotype on the risk of HTG was only evident in subjects in healthy population and not in T2DM and speculated that probably because of the existence of a strong insulin resistance in them masked the effects of genetic polymorphism [7]. Indeed, recent studies have shown that patients homozygous for the -455C APOC3 variant were poorly responsive to the APOC3-lowering effects of n-3 PUFAs [26]. CHD patients also suffered from different complications, such as T2DM, Hypertension, abdominal obesity, and Fatty liver et al, they all could mask the effect of a common APOC3 genotype on the risk of CHD. Although we excluded the population of T2DM, there was still no relationship found between them(data not shown). Maybe there was other larval factors we yet not found in CHD mask the association between APOC3 T-455C or C-482T and CHD. Such findings still have other explanations. Firstly, the number of our study samples may be too low to detect a significant association. Secondly, CHD was a multifactorial desease, one or more genetic variations combining with environmental factors may result in phenotypic variability. Thirdly, part of CHD risk attributable to elevated triglyceride levels was due to structural alterations of LDL and HDL, such as oxidation. Fourthly, race differences, genetic background, or environmental differences or methodological flaws or nonrandomized selection of subjects. Finally, a number of conditions such as age, obesity, gender, hormonal status may influence the relationship between APOC3 gene polymorphisms and CHD.

## Conclusions

Altogether with previous and present findings suggested, although APOC3 promoter variants had a key impact on plasma triglyceride and APOC3 levels, this effect didn't raise significantly CHD risk in Chinese Han Population. That was, APOC3 T-455C and C-482T SNPs were not major risk factors of CHD in Han Population in East China. However, other studies were necessary to confirm these results and search for the causing facts.

## List of abbreviations

APOC3: apolipoprotein C3; HTG: hypertriglyceridemia; CHD: coronary heart disease; TRL: triglyceride-rich lipoprotein; AS: artherosclerosis; IRE: insulin-responsive element; MCP: monocyte chemoattractant protein; IL: interleukin; T2DM: type 2 diabetes mellitus.

## Competing interests

The authors declare that they have no competing interests.

## Authors' contributions

The study was designed by RZ, JY and HW. JY, JH and YL equally contribuited to the work. All subjects data were obtained by BQ, JH and YL. The database organization was carried out by RZ and HW. Experimental data was obtained by JY and BQ. Data analyses were performed by SH and JX. The paper was written by JY and RZ and all authors read and approved the final manuscript.
